# Public engagement in decision‐making regarding the management of the COVID‐19 epidemic: Views and expectations of the ‘publics’

**DOI:** 10.1111/hex.13583

**Published:** 2022-09-23

**Authors:** Sophie Kemper, Frank Kupper, Sandra Kengne Kamga, Anne Brabers, Judith De Jong, Marloes Bongers, Aura Timen

**Affiliations:** ^1^ National Coordination Centre for Communicable Disease Control The National Institute for Public Health and the Environment Bilthoven The Netherlands; ^2^ Athena Institute, Faculty of Science VU University Amsterdam Amsterdam 1081 HV The Netherlands; ^3^ NIVEL, The Netherlands Institute for Health Services Research Utrecht 3513 CR The Netherlands; ^4^ Health, Medicine and Life Sciences Maastricht University Maastricht The Netherlands; ^5^ Department of Primary and Community Care Radboud University Medical Centre Nijmegen The Netherlands

**Keywords:** COVID‐19 epidemic, deliberative discussion focus groups, epidemic management, public engagement, public perspective

## Abstract

**Background:**

In the management of epidemics, like COVID‐19, trade‐offs have to be made between reducing mortality and morbidity and minimizing socioeconomic and political consequences. Traditionally, epidemic management (EM) has been guided and executed attentively by experts and policymakers. It can, however, still be controversial in the public sphere. In the last decades, public engagement (PE) has been successfully applied in various aspects of healthcare. This leads to the question if PE could be implemented in EM decision‐making.

**Methods:**

From June to October 2020, seven deliberative discussion focus groups were executed with 35 Dutch citizens between 19 and 84 years old. Their views on PE in COVID‐19 management were explored. The deliberative approach allows for the education of participants on the topic before the discussion. The benefits, barriers, timing and possible forms of PE in EM were discussed.

**Results:**

Almost all participants supported PE in EM, as they thought that integrating their experiences and ideas would benefit the quality of EM, and increase awareness and acceptance of measures. A fitting mode for PE was consultation, as it was deemed important to provide the public with possibilities to share ideas and feedback; however, final authority remained with experts. The publics could particularly provide input about communication campaigns and control measures. PE could be executed after the first acute phase of the epidemic and during evaluations.

**Conclusions:**

This paper describes the construction of an empirically informed framework about the values and conditions for PE in EM from the perspective of the public. Participants expressed support to engage certain population groups and considered it valuable for the quality and effectiveness of EM; however, they expressed doubts about the feasibility of PE and the capabilities of citizens. In future studies, these results should be confirmed by a broader audience.

**Patient or Public Contribution:**

No patients or members of the public were involved in the construction and execution of this study. This study was very exploratory, to gain a first insight into the views of the public in the Netherlands, and will be used to develop engagement practices accordingly. At this stage, the involvement of the public was not yet appropriate.

## 1 INTRODUCTION

In our culture, we try to control every aspect of life. COVID‐19 is so unpredictable… It affects both young and old and comes with many uncertainties. Blissful uncertainties as I like to call it. Finally, there is a break from control. And I'm not sure what we will gain from it. A crisis like this equals both danger and opportunity. (Study participant, 2020)

From December 2019 onwards, the severe acute respiratory syndrome coronavirus 2 virus, which causes COVID‐19, has spread across countries worldwide leading to a pandemic. It has heavily impacted the health and safety of citizens, as well as other aspects of society, such as the economy, social structures and politics.^1^ When an epidemic such as COVID‐19 occurs, its management is pivotal in containing the virus. According to the World Health Organization (WHO), the goal of epidemic management (EM) is: ‘to mitigate its impact and reduce its incidence, morbidity and mortality as well as disruptions to economic, political, and social systems’.^2^ EM is used in this study as an overarching term that entails the step‐by‐step process of decision‐making regarding all necessary actions before, during and after an infectious disease outbreak, to minimize the impact of the outbreak on all aspects of society.^3^, ^4^

In the urgency of EM decision‐making, various societal principles, such as solidarity, justice and liberty, have to be weighted, within a climate of fear and distress. Other characteristics of epidemics, such as social disruption and scientific uncertainty, complicate these trade‐offs even more.^5^ Traditionally, EM has been mostly guided by public health organizations, governmental bodies, and scientific experts.^6^, ^7^ Their blend of expertize and experience is used to trade‐off between reducing mortality and morbidity and minimizing its associated socioeconomic and political consequences, within troubling circumstances.^8^, ^9^ This complex interplay of principles, troubling circumstances and strong decision‐related impacts within EM raises questions about how decisions are being made. As we currently rely heavily on experts, valuable input from other sources might be overlooked, for instance, that of the public.^10^

Recently, public health officials, such as the WHO and ECDC, have been emphasizing the importance of public engagement (PE) in the management of various epidemics.^2^, ^11^ PE is the spectrum of processes and activities that brings the public into a decision‐making process. In the literature, three main rationales for PE exists.^12^, ^13^, ^14^ First, the normative rationale describes engagement itself as a valuable process that increases the democratic validity of decision‐making. Second, the instrumental rationale describes PE as a means to obtain the most beneficial outcome. Deliberation with citizens provides policymakers with information about the failure or success of certain policies. Simultaneously, citizens acquire information about the intent and context of policies, which can foster trust and understanding. Overall, both the public and policy makers can gain insight into EM from PE, which could potentially result in a more fitting course of action, mitigation of opposition to a chosen policy, and an increase in support.^8^, ^15^ This could especially be important when the public has already been showing much discontent with implemented EM policies. During COVID‐19, this happened in the Netherlands on several occasions, as many demonstrations, protests and petitions were set up by the public.^16^, ^17^ Even riots arose as a backlash to the implemented nightly curfew.^18^ Third, the substantive rationale entails using the values of the public as a foundation for policies. These values transcend interests attached to certain positions or systems. Experiential knowledge is respected in decision‐making and could complement expert knowledge.^9^, ^19^ Moreover, the public could perceive problems and solutions that experts may not notice.^20^ The desired mode of PE is context‐specific and can vary between informing, consulting, collaborating with and empowering the public.^21^

Despite the seemingly promising potential of PE, until now, a few efforts have been made to integrate the perspective of the publics (this ‘public’ cannot be classified as monolithic, but actually comprises people with a diverse range of demographic, epidemiologic, social and economic characteristics. To respect this complexity and diversity, the term ‘public’ is replaced by ‘publics’. Publics refers to all persons living in the Netherlands, with no limitations on a particular group based on demographic, epidemiologic, social or economic conditions) in EM.^22^, ^23^ Which could be an indicator of how challenging integrating PE in EM is, due to the complex nature of EM. For instance, in the United States, Mexico and Nicaragua, communities were consulted to shape culturally appropriate control strategies and communication efforts concerning Zika virus and dengue virus, which resulted in a higher‐quality EM on a local level.^24^, ^25^ Specifically in the Netherlands, valuable citizen assemblies and consultations have been executed to reveal public preferences on for instance vaccination strategies and relaxation of measures.^26^, ^27^, ^28^ However, many of these examples are one‐time engagement efforts without clear follow‐up. Besides, most of these practices are predefined, and the engaged publics to be are not asked about their preferences on the forehand. In this study, we try to take a step backwards and gain insight into the views of the publics concerning their engagement in the management of COVID‐19, to identify accompanying possibilities and challenges.

As the opening quote stated, the COVID‐19 epidemic can be seen as an opportunity to learn. This paper explores the possibilities for the role of the publics in COVID‐19 EM in the Netherlands, which leads us to the following research question:

What views and expectations on public engagement are present in the management of the COVID‐19 epidemic from the perspective of the publics in the Netherlands?

## 2 METHODS

Between June and October 2020, seven Online Deliberative Discussion Focus Groups (DDFGs) were held with members of the general public in the Netherlands. The deliberative approach leads to more knowledgeable and thoughtful participants, especially on subjects that may be somewhat unfamiliar.^29^ We expected that EM might be unfamiliar to participants. All sessions were moderated by two researchers (S. K. with F. K. or L. S. K. K.) and lasted 2 h. The online sessions were facilitated via the meeting software GoToMeeting and were executed in Dutch. The DDFGs were not intended to yield a representative sample of the Dutch population but to provide an in‐depth exploration of the diversity of views that exist among the publics. The first three DDFGs were held in June 2020, when the first epidemic wave in the Netherlands had ended and the situation had stabilized. In response, the government decided to relieve restriction measures. The second set of DDFGs (number 4–7) was held in October 2020, when the outbreak situation was deteriorating again and new restriction measures were announced.

Participants were recruited via two panels. The first three DDFGs were executed with panel members of the Dutch Health Care Consumer Panel, which is managed by Nivel, the Netherlands Institute for Health Services Research. To maintain social homogeneity in the sessions, age stratification was applied. This decision was made because age has an influence on risk perceptions and protective behaviour during the COVID‐19 epidemic.^30^ Per age category (see Table 1), a random sample was taken from the panel members (around 1500 panel members), who subsequently received an e‐mail invitation to participate. From the panel members who wanted to participate, a selection was made based on gender, age (within the designated age category), education level and place of residence, to strive for maximum diversity within all three DDFGs.

**Table 1:** Information on characteristics of DDFGs with regard to date, used panel, and age group

Number of DDFG	Date of execution	Panel used	Age group
1	04 June 2020	Nivel	46–64 years
2	05 June 2020	Nivel	18–45 years
3	10 June 2020	Nivel	65 years and older
4	01 October 2020	CG Research	18–45 years
5	02 October 2020	CG Research	46–64 years
6	05 October 2020	CG Research	65 years and older
7	15 October 2020	CG Research	18–45 years

Abbreviation: DDFG, Deliberative Discussion Focus Group.

The remaining four DDFGs were executed in collaboration with CG Research, a general market research firm. After the first three DDFGs, the research team (S. K., F. K., M. B., A. T.) decided that the views captured did not entirely correspond with the whole range of views present in the publics. This decision was based on a rough analysis of the public discourse at that period by means of news articles, and a national study concerning the attitudes and behaviour of the public. Overall, much criticism was expressed regarding the management of COVID‐19, and the publics felt that they were not being heard.^31^, ^32^, ^33^ These views did not entirely correspond with what the participants of DDFGs 1–3 expressed, as they appeared to be more satisfied with how COVID‐19 was managed at that time. To broaden the diversity of views within the sample population, a second panel was used. For the sampling procedure, stratification by age was again applied, of which a random sample was taken. The age categories per DDFG are displayed in Table 1. However, before participants were eligible, they had to react to the following two statements on a 7‐point Likert scale, which ranged from ‘1 = strongly disagree’, to ‘4 = neutral’, to ‘7 = strongly agree’:

1. My voice needs to be increasingly heard about how we are managing the COVID‐19 epidemic in the Netherlands.
2. The government needs to involve me more in decisions regarding COVID‐19 management in the Netherlands.

By utilizing these statements, people who wanted their voices to be heard were recruited. These views would hypothetically lead to different values regarding PE in EM, hereby attempting to broaden the diversity of views regarding PE in EM in the sample population. Participants who answered between the range of 5–7 on the Likert scale for both statements, were deemed eligible. A further selection was made based on gender, age (within the designated age category), education level and place of residence to strive for diversity.

### 2.1 Structure and content

All DDFGs consisted of three elements. They started with an introduction and explanation of the purpose of the study and recorded informed consent was obtained. Then, a presentation was shared about the experiences with COVID‐19 from the perspective of the Centre for Infectious Disease Control in the Netherlands, which invited participants to reflect on experiences outside their own. For example, information was shared about the activities that the Centre executed to prepare for the COVID‐19 epidemic.^34^ After this, an in‐depth discussion about PE in COVID‐19 management was held. Discussions focused on the potential benefits of PE in EM and favourable timing of PE in the epidemic. After this, participants were informed about possible modes of PE in EM based on the IAP2 framework of public participation.^21^ This framework comprises five possible modes of PE, depending on the type of interaction and power: *inform, consult, involve, collaborate*, and *empower*. The various modes of PE were explained by an example that had no link with EM (Table 2). It was stressed that one mode of PE was not better than other modes, as this entirely depends on the context. Subsequently, desirable modes of PE in EM were discussed. The two presentations are what created the deliberative nature of the DDFGs.

**Table 2:** The five modes of public engagement with explanations given to the participants during the DDFGs

Inform	Consult	Involve	Collaborate	Empower
The public is provided with timely and consistent information.^a^	The public is asked for feedback on questions or problems. This feedback is nonbinding.	The public is asked for advice for the whole process, and their advice is integrated into the final decisions.	The public is seen as a partner.	The public has the ultimate decision‐making power. They receive support.

^a^ The first mode of engagement presented was *inform*, which is in itself not necessarily a mode to actively integrate the perspective of the other. Nevertheless, it was important to mention as in some situations, there may be no need for active engagement.

### 2.2 Data analysis

The recordings of the sessions were transcribed verbatim, and a thematic analysis was executed using MAXQDA 2020 software. The thematic analysis approach, which used both inductive and deductive coding, was chosen to identify, organize and reveal patterns of meaning derived from the content of the data itself.^35^ Two researchers (S. K. & L. S. K. K.) separately coded DDFG 1. The differences and similarities were analysed (S. K.), discussed (S. K. & L. S. K. K.) and improved (S. K. & L. S. K. K.). This process was repeated for both DDFG 2 and DDFG 3. Finally, the final codebook was discussed and agreed upon (S. K., L. S. K. K., J. F. H. K.). The final codebook of the first three sessions was subsequently used for thematic analysis of the second set of sessions while allowing for emerging codes. The research team (S. K., F. K., M. B., A. T.) decided to divide all categories into the following themes: *Why, How, When* and *Who*. These themes fitted the data as these suited the content of the categories and the goal of the study, to build a foundation for PE in EM. To identify differences between the two groups of participants and the period of execution, newly emerging themes from the second set of DDFGs were emphasized.

The study protocol was approved by The Centre for Clinical Expertise at the National Institute for Public Health and the Environment (study protocol number LCI‐445).

## 3 RESULTS

### 3.1 Participants characteristics

In total, 35 citizens participated in seven online DDFGs. Every session had 4–6 participants, which was suitable for the online nature of the sessions. The characteristics of the participants are displayed in Table 3.

**Table 3:** Composition of the seven DDFGs

Composition of deliberative discussion focus groups								
Characteristics	DDFG 1	DDFG 2	DDFG 3	DDFG 4	DDFG 5	DDFG 6	DDFG 7	All (%)
Participants (*n*)	5	4	6	4	5	5	6	35
Age range in years	54–64	35–42	67–84	19–42	48–56	65–72	19–33	19–84
Gender								
Female	2	3	2	2	3	2	3	17 (49%)
Male	3	1	4	2	2	3	3	18 (51%)
Education^a^								
Level 0–2	0	0	0	1	1	0	0	2 (6%)
Level 3–4	4	1	0	2	2	2	3	14 (40%)
Level 5–8	1	3	6	1	2	3	3	19 (54%)
Region of residency								
North	0	0	0	0	0	0	0	0
East	0	1	1	1	3	0	2	8 (23%)
South	0	1	4	2	0	0	0	7 (20%)
West	5	2	1	1	2	5	4	20 (57%)
Range of answers on the Likert scale	–	–	–	6–7	5–6	5–7	5–6	5–7

*Note*: All values are given in absolute numbers.

Level 0–2: Early childhood education, primary education or lower secondary education.

Level 3–4: Upper secondary education or postsecondary/nontertiary education.

Level 5–8: Short‐cycle tertiary education, bachelor's (or equivalent), master's (or equivalent) or doctoral (or equivalent).

Abbreviation: DDFG, Deliberative Discussion Focus Group.

^a^ Education is classified by The International Standard Classification of Education (ISCED) by UNESCO, 2011.

### 3.2 PE in COVID‐19 management

#### 3.2.1 Why and why not: Values regarding PE in EM

Most participants stated that their current role in EM mostly entails receiving information about COVID‐19 management (see Table 4 for supporting quotes for this theme). The majority of the participants believed that it would be beneficial for the quality of EM to increase the engagement of the publics. Several arguments for this were given, as well as reasons against engagement. First, some participants believed that being engaged in EM is a citizen's right. They found that the government should listen to citizens and take their perspectives seriously in policy‐making. According to one participant, the feeling of being ‘listened to’ would contribute to a sense of solidarity. More participants agreed that this would subsequently decrease public unrest and dissatisfaction of the publics with the final policy. Second, some participants found that the publics could provide beneficial information for policy makers to take into account such as ideas to solve problems that experts encounter. Participants identified three areas: the public could provide insight into differences in the needs of various population groups; the public could contribute through personal expertize of certain citizens and could aid in the translation of policies to day‐to‐day lives. Third, some participants stated that awareness and understanding are needed in the whole process of decision‐making in EM: Why and how the decision was made? According to the participants, by actively engaging the publics, awareness and understanding of EM policy will increase, which will create support for restriction measures, as well as lowering public unrest.

**Table 4:** Supporting quotes for results about why and why not: Values regarding PE in EM

‘It is not the case that I, myself, am involved in the decision‐making … They [the government] didn't ask me anything. Measures are imposed on me’. (Female, DDFG 2)
‘I believe the chances lie in the restriction or relaxation of measures for specific groups. For example how to treat the elderly, because I think they should be treated differently than youngsters. In my opinion, this has to be different … How hard that may be. But you can talk to the people on how to achieve this’. (Male, DDFG 4)
‘If you [a member of the publics] can think along, you will also receive more information. You are more knowledgeable and there is foundation for a real discussion’. (Male, DDFG 5)
‘I think listening to the publics would be good. However, I don't think it is achievable when swift action is necessary’. (Male, DDFG 2)
‘We live in a free country with many different opinions, so there will always be people complaining that their opinion was not heard. As such, there will always be dissatisfied people’. (Female, DDFG 7)

However, participants mentioned several reasons not to engage the publics. Time constraints were mentioned as a big challenge to PE, which illustrated the assumption that PE is time‐consuming. Second, for many participants, it was almost impossible to imagine how to engage and summarize hundreds of opinions and filter out unrealistic suggestions. Furthermore, some participants stated that not all opinions of the publics can be incorporated into EM, simply because there are so many. This could again lead to dissatisfaction and affect the support base for the final policy.

#### 3.2.2 How: Process of PE in EM

When discussing the five modes of PE, *inform* was mentioned most frequently. Most explicitly stated that informing the publics on the EM policy is essential (Table 5). They felt that *inform* was currently executed the most and expressed much criticism on the current manner of execution. The current provision of information did not support the questions and needs of the participants. Most criticisms were expressed on the communication of the government, which was viewed as inconsistent, vague, complicated and negatively framed. Furthermore, a few participants stated that they felt overwhelmed by the information overload online at the start of the epidemic. Other participants also held the media accountable for unclear information. According to the participants, the inadequate communication resulted in public unrest, lack of confidence in the government and decreased compliance with restriction measures.

**Table 5:** Supporting quotes for results about how: Process of PE in EM

‘In my opinion, informing is not only essential, but also a major obligation that the government has’. (Female, DDFG 6)
‘The information is very inconsistent. One person claims the utility of facemasks, whereas an expert in America claims it only works under certain conditions, and another expert claims it is total nonsense. This happens for all kinds of restriction measures’. (Male, DDFG 6)
‘During a pandemic, you should not give the publics all the power because you need a strong hand in this … I think that eventually the solution lays somewhere between consult and involve, because people will feel heard, and ideas from the publics can be used to improve certain aspects of EM’. (Male, DDFG 3)
‘The educational federations should be authorized to make a statement towards the government about how to arrange affairs in schools. These federations are responsible for individual schools. And the schools have to align with parents and children’. (Male, DDFG 2)
‘I don't agree with “empower”, because it has such a big effect on our country and the rest of the world. Due to the scale, I think empower is really not suitable’. (Female, DDFG 5)
‘What you need as a citizen is the ability to trust the people with the most critical positions in decision‐making. Trust is only possible if they make it clear why certain decisions were made, and what they would do differently in the future’. (Male, DDFG 3)

Abbreviations: EM, epidemic management; PE, public engagement.

According to almost all participants, the most fitting mode of PE in EM would be *consult*. It was important for them that the publics receive more influence in EM. The participants explained that with *consult*, the publics feel that they are being listened to and taken seriously, and they can provide ideas to improve the quality of EM. Participants stressed that experts should still be in the lead. Participants found it valuable to let the publics function as a sounding board, to provide decision‐makers with insight into their experiences. Feedback from the publics could be asked to prevent unclarities in communication efforts. A few participants, however, found that consultation was already taking place, due to the existence of the representative democracy in the Netherlands. As such, the representative of their choice in the government already represented their perspective. Subsequently, they did not feel the need to increase engagement.

Less frequently mentioned, but still possibly a fitting mode according to participants was *involve*. The participants expressed two benefits of this mode of engagement: (1) PE during the whole process of EM instead of only during specific problems and (2) mandatory instead of voluntary incorporation of the publics' contributions (in *Involve, Collaborate* and *Empower*, the perspective of the publics is binding for final decision‐making). A few participants suggested to *involve* some kind of representative of the publics in EM. This representative would portray the needs, attitudes and knowledge levels of the rest of the publics. Another suggestion was to involve sector associations together with members of the publics for EM decisions within their sector.

*Collaborate* and *empower* were deemed not suitable by most participants because of the publics' lack of knowledge and experience with EM. This undermined the trust they had in the capabilities of the publics to contribute. All participants agreed that providing the publics with the decision‐making power in EM would not be desirable. People expressed suspicions that those engaged might put their own interests ahead of the interests of the general public.

Overall, many participants found it meaningful to receive more details about why certain decisions were made. In addition, when integrating PE in EM in practice, it would be important to hear why the views of the publics would be integrated or why not. In line with this, some participants felt a lack of transparency in EM from the government. They expected that increased transparency leads to increased trust of the publics in the government. This trust was deemed crucial during an epidemic by a few participants.

#### 3.2.3 When: Period for PE

According to most participants, the priority at the start of an epidemic is to rapidly control it. At that stage, swift action is necessary and a lack of knowledge is likely. Due to these beliefs, PE was deemed not beneficial at the start of the epidemic (Table 6). Most participants felt that the virus was something that did not greatly affect them at the start. They did not yet realize the severity of the situation. Most participants suggested that at a stage when the epidemic would be more controlled and more time is available, the publics could be engaged more. Some participants argued that later in the epidemic there would have been more room to customize relaxation/restriction measures for different population groups. Some stated that PE should only be executed after the epidemic to use the feedback for the next epidemic. One idea was to integrate the experiences of the publics with communication efforts into specific epidemic scripts.

**Table 6:** Supporting quotes for results about when: Period for PE

‘It depends on how controllable the situation is. It was not in the start of the epidemic, so there was no time to extensively discuss, and provide the publics with the needed knowledge. Because when you want to engage the publics, you have to create some depth into the knowledge. As such, when there is a sense of urgency, just do inform. Later, you can think about consult and after that … But at that point, the situation is already in control and you can afford to discuss it with multiple people. The first phase needs speed’. (Female, DDFG 5)
‘In the next phases you can engage the publics, but in first instance it is important that the experts, in this case with the COVID‐19 epidemic, decide how to manage it. First decide what the virus is and what it does etcetera. And after this you can look for opportunities to think along’. (Female, DDFG 2)
‘We can evaluate what went right and what should be done differently the next time, and create scripts based in this evaluation. So when a similar situation occurs, you will not be overwhelmed’. (Male, DDFG 1)

Abbreviation: PE, public engagement.

Participants believed incorporating PE in EM would require time and effort. Two aspects contributed to this impression. First, the participants agreed that the publics lack any pre‐existing knowledge about EM. Before they can be engaged and make a serious contribution, they should also be informed about the virus and EM process. Second, participants found the organization of the PE process time‐ and energy‐consuming. This refers back to the high number of opinions of the publics one would have to collect and consider.

#### 3.2.4 Who: Characteristics of engaged publics

All participants agreed that people who have the most knowledge about and training in EM should have the ultimate decision‐making power. Furthermore, participants stated that these experts are more capable of making decisions for the sake of public gain instead of personal gain. The suggestion to engage sector organizations as representatives instead of individual citizens was made by multiple participants (Table 7). They believed that these organizations can provide clear insight into the problems and solutions within their specific sector concerning control measures as they have the most knowledge about what does and does not work in their sector. Participants gave sector examples, including hospitality, entrepreneurs, gyms, education, hairdressers and nursing homes.

**Table 7:** Supporting quotes for results about who: Characteristics of engaged publics

‘I am sure that every sector has solutions for the problem. A problem arises and every sector can find their own solutions. The ministries should not come up with these solutions, you have to let the people figure it out themselves’. (Female, DDFG 2)
‘I think that if you want to engage everyone, the ones who scream the loudest will get their way, which is what is happening now. If a minority thinks we should do A, and the silent majority thinks we should do B, A will be implemented because of the fuss. I think the only possibility is inform’. (Female, DDFG 1)
‘Conspiracy theorists will say; this is not necessary, and that should not happen, and this is wrong…. They are going to interfere with aspects they think they know about, but in reality do not have any knowledge on’. (Female, DDFG 5)
‘I am still thinking about two groups within the public. You have the people who are analyzing everything, who are considerate, who are sensible and who can make correct conclusions. And you have the sheep, who do not understand everything well but who are constantly stomping their feet. And of these groups who do we have most in society? … I can conclude that these people are a big part of society. If you can calm them… but then again, this is a dangerous statement as I am judging myself, which is also not correct’. (Female, DDFG 5)

When focusing on the diversity of the publics, a few comments were made. In general, the participants made a division in the publics based on differences in risks and interests. The division was based on characteristics, such as age, profession, risk of COVID‐19, education level, ethnicity and people with certain beliefs on science such as conspiracy thinkers. Doubts about the capabilities of the latter group to engage were expressed, as they could lack awareness about dangers and risks regarding EM according to the participants. Also, some concerns were raised about counterproductivity if publics are engaged and openly avert measures that need to be taken.

#### 3.2.5 Differences between the first and second set of DDFGs

The differences in recruitment resulted in differences in the level of criticism expressed about current COVID‐19 management. During DDFGs 4–7, participants expressed more criticism and less trust in governmental bodies. A higher need for transparency was voiced by these participants, compared to DDFGs 1–3. Besides, these participants expressed a decreased satisfaction with the status quo of EM in DDFGs 4–7 and an increased need for PE. These attitudes might be partly explained by the different timing and context of DDFGs 4–7. However, the general attitude of the two groups towards the five modes of PE did not seem to differ. In addition, no new values regarding PE in EM emerged.

## 4 DISCUSSION

We constructed an empirically informed framework based on the views of the participants about *why or why not* to include the publics in EM, *how* this might be done, *who* to engage and *when* this could best be implemented in the management of COVID‐19. *Consult* was believed to be the most suitable mode of engagement and the experts in EM should maintain the final decision‐making power. Overall, this is the first exploratory study to reflect upon this type of insight into PE in COVID‐19 management from the perspective of the publics. Moreover, the data were collected during the COVID‐19 epidemic, which yielded relevant outcomes of current interest.

### 4.1 Principal findings

Overall, participants expressed positive attitudes towards PE in EM. From their perspective, engaging certain groups of the publics in the Netherlands could potentially lead to a decrease in public unrest and dissatisfaction, an increase in valuable ideas for and contributions to decision‐making, and an increase in awareness and the support base for control measures and policies in general. In other studies about PE in the specific context of epidemics and the COVID‐19 pandemic, the importance and added benefit of engaging the publics in various aspects of EM are extensively debated, from the perspective of professionals.^19^, ^36^, ^37^, ^38^, ^39^ Various authors have made policy recommendations to engage the publics early in the planning of appropriate control strategies, to make strategies more feasible and acceptable.^8^, ^22^, ^40^, ^41^ Furthermore, by PE, improvements in communication efforts could be identified.^24^, ^42^, ^43^, ^44^ From the perspective of publics themselves, two studies incorporated PE in EM and evaluated this afterwards with their participants. They found comparable benefits to PE in EM according to participants. For instance, Mouter et al.^28^ engaged the publics in the Netherlands on their preferences concerning possibilities for relaxing lockdown measures during COVID‐19. More than half of the participants indicated that they learned more about the dilemmas that the government faces. The Public Policy Centre of the University of Nebraska (2007) engaged the publics in deliberations about trade‐offs between social and economic aspects and control measures, to slow the spread of an Influenza pandemic. For participants, PE resulted in a feeling of inclusion and being listened to. Participants also believed that engagement would increase the support for the final decision.^23^

On the other hand, relevant drawbacks of PE in EM were pointed out by our participants. These included (1) time constraints due to urgency; (2) the impossibility to engage all different existing opinions and filter out unrealistic suggestions; (3) the lack of knowledge of EM in the publics and (4) personal biases of the publics. Time constraints, difficulties regarding the process and personal biases are drawbacks that we see more often in the PE literature.^14^, ^41^, ^45^ Suggestions to overcome these include integrating courses on PE in academia to reduce extra time and capacity building to deliver PE activities, focusing on the experiential knowledge of publics instead of expert knowledge, and building structures and partnerships with communities in the preparedness phase. This will increase public trust, decrease cynicism in the PE process, and reduce the time investment needed during the epidemic.^46^, ^47^ With regard to the expressed issues on how to handle the different existing opinions amongst the publics, natural language processing and active learning techniques could be used.^48^, ^49^ For instance, Liscio et al.^50^ used these techniques to identify a list of values concerning lifting COVID‐19 measures in the Netherlands. All comments made during engagement could be used to create a value list concerning the designated topic to take into account by policy‐makers. Participants also suggested only engaging groups within the public who are concerned with the specific subject of decision‐making, such as sector organizations for gyms with regard to restriction measures in gyms, or engaging youngsters about specific restriction measures. Engaging specific groups for a specific subject within EM could aid in the practicability of PE. At the same time, some participants considered certain population groups less fit for PE, mainly aiming at those who openly avert all kinds of measures to control COVID‐19, as this would be counterproductive. However, excluding people from PE in EM based on their beliefs will disregard the representativeness of such engagement processes. This could lead again to even more dissatisfaction and distrust by these excluded groups.^51^, ^52^ To determine how to handle this, further research needs to be done about for instance the magnitude of these groups within the Netherlands.

According to our participants, improvements are needed in the accountability and transparency of decision‐makers in COVID‐19 management, which is supported by similar findings in other contexts, such as priority setting in healthcare, environmental issues and governance practices.^53^, ^54^, ^55^ Survey data from the Netherlands, obtained between 17 April 2020 until 28 November 2021 showed that the positive attitude of the respondents towards their trust in the government decreased throughout the epidemic, from 73% to 16%. PE can be a means to increase this trust.^56^ Litva et al.^57^ identified a possible mode of PE, accountable consultation: ‘contribution to decisions by expressing views, a guarantee that this contribution will be heard, no responsibility for the decision but an explanation of the rationale for the decision ultimately made’. This is in line with the general desires we found in our participants: to potentially make useful contributions, to feel guaranteed to be heard, to not have the final responsibility and to receive feedback on why their contributions are reflected in the final policy or not. At the start of the epidemic especially, PE in EM was deemed less appropriate by participants. This could be related to the low sense of urgency that was felt at the beginning. The threat felt far away from people, both mentally and geographically, which impacted their personal degree of involvement. Risk communication from the government could contribute to issue formation to encourage publics to be engaged at an earlier stage. Of course, EM remains complex, and a possible lack of knowledge and evidence may continue to exist.^58^, ^59^ Altogether, there are clearly specific aspects of EM that remain challenging, and which explain the views of the publics with regard to the extent to which PE can be incorporated into EM. These should be taken into account when doing this in practice.

## 5 LIMITATIONS

Multiple characteristics of the DDFGs could have impacted the attitudes of the participants, such as socially desirable behaviour in a group setting, influence of facilitators and information that was provided. To combat this, multiple strategies were implemented such as creating an open context, limiting the number of participants and establishing rapport, which was occasionally difficult to do because of the online nature of the DDFGs.^29^ With regard to our study population, no persons living in the north of the Netherlands were included, persons with an education level between 0 and 2 were underrepresented and no stratification for ethnicity was applied. This is unfortunate, as these groups could have experienced the epidemic differently; for instance, there were fewer COVID‐19 cases at the time of the DDFGs in the North and people with a migration background in the Netherlands suffered more health and societal consequences from COVID‐19.^60^, ^61^ We are aware that our sampling strategy could have led to a sample population with two opposing views on PE in EM, and might not be comparable to each other. Besides, participants of DDFG 4–7 might have biased the overall results towards a less critical view of PE in EM. Furthermore, results might have been different if we did not use panels, as these populations differ in certain characteristics from the general population, for instance, they might display a more positive attitude towards engagement, if they regularly attend focus groups to share opinions. Regarding the study context, the future course of the epidemic was uncertain, these feelings of uncertainty and fear could have impacted the attitudes of the participants, mostly on the sense of urgency about PE in EM. These participants could have graded PE in EM as less important in hindsight, knowing that the epidemic is over. On the other hand, recall bias is minimized.^62^ Overall, it is important to keep this context in mind, and the fact that the study is conducted during two different time periods in the outbreak.

### 5.1 Future research

To our knowledge, this is the first study that directly explores the views of publics on possibilities for PE in EM. The next step would be to identify the views, expectations and needs of various groups within the publics. ‘The public’ is not a homogeneous entity but a complex and dynamic collection of multiple groups with various characteristics. This could impact the approach to PE in EM and its diversity. In line with this, more attention should be given to conceptual clarification of the various groups within the publics who can contribute to EM decision‐making such as the representatives our participants suggested, and be aware of inclusivity and diversity within these groups.

## 6 CONCLUSIONS

This paper explored the perspective of the ‘publics’ on PE in decision‐making regarding the management of the COVID‐19 epidemic in the Netherlands. This exploration was done in the midst of the COVID‐19 epidemic itself, which was a unique opportunity. The participants agreed that targeted PE could positively influence the quality and effectiveness of COVID‐19 EM. Furthermore, the participants called for more accountability of the decision‐makers, and more transparency in the EM decision‐making process.

As our participants are clearly aware of the complexity of EM, they are not asking to replace current decision‐makers in EM. What they do wish is for their voices to be heard and their experiences, ideas and feedback to be taken seriously in developing and improving COVID‐19 management.

## Data Availability

The datasets used and/or analysed during the current study are not publicly available due to data protection and confidentiality. Data are however available upon reasonable request from the authors and with permission of the Centre for Infectious Disease Control in the Netherlands.
